# Cross-cultural adaptation and psychometric evaluation of the individual workload perception scale-revised (IWPS-R) in Spanish nurses

**DOI:** 10.1016/j.ijnsa.2026.100517

**Published:** 2026-03-03

**Authors:** Juan David Fernández-Sánchez, Fernando Andrés-Pretel, Inmaculada Pérez-Rodríguez, Milagros Molina-Alarcón, Natan Redondo-Perez, Mercedes Fernández-Castro, Cristina M. Lozano-Hernández

**Affiliations:** a Faculty of Nursing of Albacete, University of Castilla-La Mancha, 02071 Albacete, Spain; b University Hospital Complex of Albacete, C/ Hermanos Falcó n°37. 02006 Albacete. Spain; c Research Network on Chronicity, Primary Care and Health Promotion -RICAPPS- (RICORS), Spain; d Institute de Investigación Sanitaria Gregorio Marañón (IiSGM), Madrid. CIBEREHD, Spain; e Institute of Biomedicine of the University of Castilla-La Mancha (IB-UCLM), Albacete, Spain; f University Clinical Hospital of Valladolid, C/ Avda. Ramón y Cajal s/n. Valladolid. Spain; g Department of Methodology and Statistics, National Paraplegic Hospital Foundation, Toledo, Spain; h Atlantic Mediterranean Technological University, Málaga, Spain

**Keywords:** Nursing staff, Scale validation, Perception, Workload, Work environment, Hospitals

## Abstract

**Objective:**

To adapt the Individual Workload Perception Scale-Revised (IWPS-R) into Spanish and evaluate its psychometric properties among nursing professionals.

**Methods:**

We performed a cross-cultural adaptation following Beaton’s protocol, including translation, back-translation, and expert committee review. A multicenter study was conducted with 305 nurses from two Spanish hospitals. We employed Exploratory Factor Analysis (EFA) and Confirmatory Factor Analysis (CFA) using the robust Diagonally Weighted Least Squares (DWLS) estimator on a polychoric correlation matrix to address the ordinal nature of the data.

**Results:**

The final Spanish version (IWPS-R-ES) resulted in 25 items distributed across five factors: Peer Support, Unit Support, Manager Support, Workload, and Intent to Stay. The CFA supported a robust model fit: Comparative Fit Index (CFI) = 0.985, Tucker-Lewis Index (TLI) = 0.982, and Root Mean Square Error of Approximation (RMSEA) = 0.052. Standardized factor loadings exceeding 0.4 confirmed the convergent validity after removing one item with insufficient shared variance. Reliability analysis demonstrated high internal consistency (Cronbach’s alpha: 0.81–0.93; McDonald’s omega: 0.82–0.94). Correlation patterns supported the discriminant validity, confirming the scale accurately distinguishes between different dimensions of workload perception.

**Conclusions:**

The IWPS-R-ES is a valid and reliable 25-item instrument for assessing subjective workload among Spanish-speaking nurses. It provides healthcare managers with a rigorous tool to identify organizational stressors and implement strategies to improve nurse well-being and patient safety.

**What is already known**

• Poor work environments increase clinical errors and patient safety risks.
• Objective metrics fail to capture individual perceptions of demands and resources.
• The IWPS-R measures basic needs and professional well-being based on Maslow’s theory.

**What this paper adds**

• First Spanish scale to quantify subjective nursing workload.
• Validates five key dimensions to guide targeted management interventions.
• Links perceived effort to organizational health and patient safety.

Alt-text: Unlabelled box dummy alt text

## 1 Introduction

Healthcare systems are essential for achieving health objectives and ensuring safe, high-quality care. Nevertheless, the nursing profession is characterized by high levels of occupational stress, as professionals are continuously exposed to psychosocial risks with physical, psychological, and social repercussions. This impact is attributed to multiple factors, including workload, interaction dynamics and interprofessional conflicts, rigid shift schedules, scarcity of material, technical, and human resources, as well as the emotional demands intrinsic to direct patient contact (Hermansyah et al., 2019). A direct correlation has been established between increased workload and professional deterioration, which can compromise the effectiveness of nurses' performance (Liu et al., 2021).

We should raise awareness about the importance of adjusting nurses' workloads, since a high workload contributes to an increase in adverse events, nosocomial infections, and worse health outcomes for the patients they care for (Assaye et al., 2021; Ayuso-Fernández et al., 2021). The relationship between inadequate workload (or insufficient staffing) and adverse patient outcomes is strongly supported in hospital settings, as a correlation has been demonstrated between higher nurse-to-patient ratios and the incidence of hospital-acquired pressure injuries (HAPIs) and mortality. Therefore, a clear correlation is established between workload and patient safety (Fernández-Sánchez et al., 2025).

Nursing workload encompasses the time dedicated to care, the environment, and professional development, making it essential to measure and organize resources accordingly. This measurement impacts the safety, effectiveness, and well-being of both patients and professionals (Alghamdi, 2016; Ferramosca et al., 2023). High workloads can be associated with a nursing shortage, which poses a challenge for healthcare organizations but also an opportunity to transform care models. It is key to invest in technology, training, and strategic plans that value experience and support new generations (Vozzella and Hehman, 2023).

Nursing shortages and unhealthy work environments lead to inadequate care, increased absenteeism, and professional dissatisfaction. Absenteeism is related to psychosocial factors such as a lack of clarity in responsibilities, the work environment, and the organization. A poor work environment directly affects patient safety and the quality of care. In fact, it is estimated that 22 % of healthcare errors are linked to the work environment. A healthy environment with good management and collaboration improves outcomes and patient care (Al Ismail et al., 2023; Aslan and Gokdemir, 2019; Chaneliere et al., 2018). However, an organization may have excellent nursing practice environment policies (e.g., training, collaboration), but if the professional is overloaded, the workload prevents those structural resources from effectively translating into high standards of care. Nursing workload, which encompasses the volume and complexity of care demands, the time required, and cognitive effort, should not be considered an isolated variable, but as a fundamental dimension of the professional practice environment (Nursing Practice Environment). Effective workload management is, therefore, the leverage point for improvements in the nursing practice environment to impact patient safety and quality (Chiminelli-Tomás et al., 2025).

Managers should prioritize reducing workload and aligning staffing levels with healthcare needs. Moreover, understanding nurses' perceptions is essential for enhancing individualized care (Driscoll et al., 2018; Pamuk and Özyürek, 2022).

Lack of support and workload overload lead to dissatisfaction among nurses, which also impacts patients and hinders staff retention. Measuring workload objectively alone fails to consider key factors such as experience, organizational support, or available resources (Fuentelsaz-Gallego et al., 2013).

In the U.S., the Individual Workload Perception Scale (IWPS) was developed to measure workload perception in specific pediatric contexts (Cox et al., 2007). British nurse Karen Cox developed the IWPS scale, based on Maslow's theory, with five dimensions: managerial support, peer support, unit support, workload, and intention to stay. This scale focuses on measuring basic needs, including physiological, safety, and social needs. According to this approach, professional development can only be addressed when these needs are met. The IWPS provides a valuable tool for enhancing the work environment and nursing job satisfaction. The nurse manager support dimension assesses staff perceptions regarding leadership availability and responsiveness to professional needs. The peer support dimension quantifies the quality of interprofessional interactions and mutual backing among nursing team members. The unit support dimension evaluates the perceived availability of essential material, technological, and infrastructural resources for the execution of nursing care functions. The workload dimension comprises indicators that quantify the prevalence of care pressure and operational overload within the nursing work environment. Finally, the intention to stay dimension seeks to measure the likelihood that nurses will remain in their jobs. The choice of instrument is based on its suitability for measuring the perception of workload and its impact on the nursing work environment.

The validated version of the IWPS, consisting of 32 items, was formally published by Dr. Karen S. Cox in 2007, where the psychometric properties of the scale were established for generalized use (Cox et al., 2007; Lacey et al., 2007). In 2010, this version was revised to optimize and refine the original instrument, forming the IWPS-R and reducing the items to 29(Cox et al., 2010) . This instrument has been widely used since its publication in the United States, with strong indicators of validity and reliability (Alghamdi, 2016; Gu and Zhang, 2014).

In the Spanish National Health System (SNS), workload management faces unique structural challenges related to chronic staff shortages in certain areas and centralized management models. The ability to accurately assess the individual perception of workload is essential for making informed decisions regarding staffing, improving the work environment, and ensuring care quality, aligning with European guidelines for patient safety.

In Spain, to date, there is no specific validated tool that assesses workload from the professional's psychometric and cognitive perspective. Although translated and utilized tools exist, such as the PES-NWI (Practice Environment Scale of the Nursing Work Index), these predominantly evaluate the practice environment (leadership, resources, and quality) or staffing (RNA4CAST). Our justification focuses on the fact that, while the PES-NWI is valuable, it does not directly measure the individual perception of demand and effort (subjective workload), which is the focus of the IWPS-R. Therefore, the validation of the IWPS-R is required to address this specific diagnostic need not covered by existing scales, justifying the necessity and relevance of validating the IWPS-R scale in Spanish (Fuentelsaz-Gallego et al., 2013; Sermeus et al., 2011).

The IWPS-R scale has also been translated and validated for other languages, namely Mandarin, Greek, Turkish, and Portuguese. The studies were conducted in general hospitals and presented very good levels of reliability of 0.93, 0.88, 0.93, and 0.88, respectively (Alikari and Fradelos, 2021; Cabrita et al., 2022; Lin et al., 2011; Özyürek and KILIÇ, 2022).

## 2 Objective

The primary objective of this study is to culturally and linguistically adapt the IWPS-R and validate its psychometric properties in the Spanish nursing population.

## 3 Methods

A multicenter, descriptive, quantitative, and cross-sectional study was conducted to adapt and validate the Revised Individual Workload Perception Scale. Data were collected from a cohort of nurses from the Spanish Public Health System of Castilla-La Mancha and Castilla y León, specifically the University Hospital Complex of Albacete and the University Clinical Hospital of Valladolid, both tertiary care units. Professionals holding positions of responsibility, such as supervisors, coordinators, or nursing managers, were excluded from the study. This exclusion is based on the need to avoid role bias and ensure the homogeneity of the construct being evaluated, since nursing management positions are, by definition, the object of evaluation for at least one dimension; including them would introduce a bias. The purpose of the instrument is to measure the workload of those providing direct care. Excluding managers ensures that the results reflect the experience of the clinical nurse and not that of the manager.

**Fig. 1:**
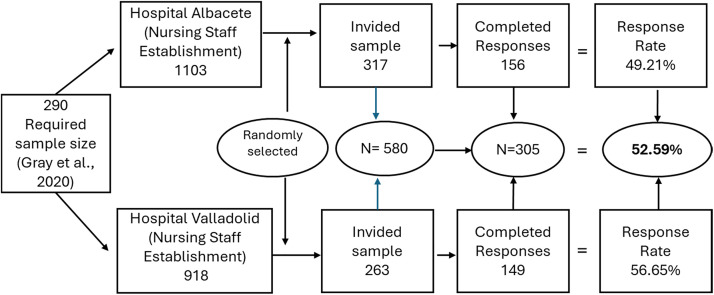
Flowchart of participant recruitment and response rates.

Data collection was conducted through a self-administered questionnaire that included the Revised Individual Workload Perception Scale. In addition, sociodemographic and occupational variables (age, sex, marital status, dependents, employment relationship, work area, seniority, and work schedule) were included in the questionnaire due to their relevance to this study. The project has received a favorable report from the Research Ethics Committee for Medicines of the Albacete Integrated Healthcare Management, with internal code No 2024–110 and dated July 18, 2024. The questionnaires were sent to the nurses' institutional email addresses and were available from 10 February to 25 March 2025. For the Final Validation phase of the instrument, a Random Probabilistic Sampling method was employed, generating a list of 580 nurses (317 from Albacete and 263 from Valladolid), who were invited to participate via email. A unique QR code leading to the online questionnaire was incorporated for each participant, where they accepted participation in the study on the first page. The number of completed questionnaires was 156 from Albacete and 149 from Valladolid, and the overall response rate was 52.59 %. The recruitment process and response rates for both hospitals are detailed in Fig. 1.

### 3.1 Instrument

The IWPS-R is a self-administered questionnaire composed of a total of 29 items, assessed on a five-point Likert-type scale (1 = strongly disagree to 5 = strongly agree) and five dimensions of nurses' perceptions of the work environment: 1. Managerial support (support from their management and middle managers, eight items); 2. Peer support (support from nurses for each other, six items); 3. Unit support (support from the supplies, resources, and services they need, six items); 4. Workload (perceptions of workload, six items); 5. Intention to stay (reflects the outcome of nurses' perceptions of workload and measures the tendency to remain on the job, five items). The total score for the results can range from 29 to 145 points, with a higher score indicating a higher level of the nurse's satisfaction with their workload (Ross, 2017).

The questionnaire takes approximately 3 to 6 min to complete.

### 3.2 Data analysis

To assess content validity in terms of translation and cultural adaptation, the methodology recommended by Beaton et al. (2000) which remains the reference protocol (the 'gold standard') for the cross-cultural adaptation of measurement instruments in the health field, despite technological advancements, due to its systemic rigor and conceptual focus (Beaton et al., 2007, 2000). The translation of the scale from English into Spanish (Castilian) was performed by two translators: an expert lay linguistic translator and a bilingual Spanish nurse. Subsequently, a second bilingual translator (English) and the bilingual Spanish nurse conducted a back-translation of the translated Spanish version. At this stage, we observed that the 29 questions were translated into English and retained the same meaning as the initial statements.

Subsequently, a synthesis or harmonization phase was carried out, where the initial translators met with a member of the research team to compare, reconcile discrepancies, and elaborate the first Spanish version of the scale.

The first version of the scale (pretest or pilot test) was administered to a sample of 20 nurses, 10 from the Valladolid Clinical University Hospital and 10 from the Albacete University Hospital Complex, using a convenience sample. The nurses read the questionnaire and were asked whether it was comprehensible (cognitive interview process). In cases of unclear or difficult points, nurses were asked to provide alternative wording without altering the meaning (informative cognitive review). Furthermore, a consensus meeting will be held following the evaluation by both groups (H. Valladolid and H. Albacete), where their proposals will be integrated into the second reconciliation version and will determine the final Spanish language version produced by the principal investigator and the bilingual Spanish nurse.

The expert version was agreed upon in a virtual meeting on November 20, 2024, with representatives from the two groups of nurses from both hospitals and the research team. For each discrepant item, a consensus version was adopted by simple majority vote, integrating the proposals to establish the third culturally adapted version of the instrument for psychometric validation.

**Fig. 2:**
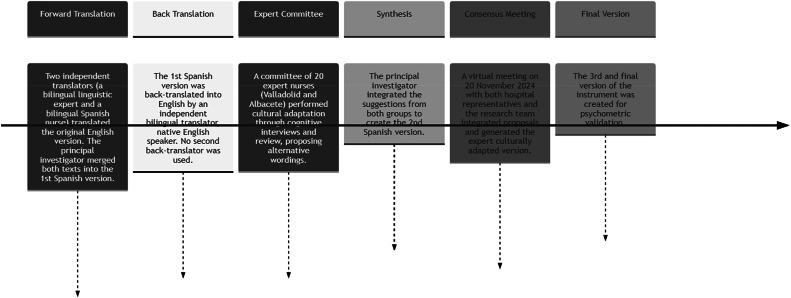
Cultural adaptation process of the instrument IWPS-R.

Using this method, we were able to conclude that the initial translations were faithful to the author's explanations and thus proceed to the validation. See Fig. 2.

For the cultural adaptation of an instrument, according to Gray et al., 2020, and for scientific expression, the sample size should be 10 individuals per item (Gray and Grove, 2020). Therefore, the minimum sample size for our study should be at least 290 participants. To compensate for potential losses, an additional 50 % was invited to the minimum required sample, resulting in a total of 580 invitations sent by random assignment.

A homogeneity analysis was conducted comparing the key sociodemographic variables between the two workplaces using the Student's *t*-test and the Mann-Whitney U test (for non-normal variables) and Pearson's χ^2^ test (for categorical variables).To ensure transparency and quality in reporting the reliability and the cross-cultural adaptation process, the manuscript has been prepared following the guidelines of the GRRAS (Guideline for Reporting Reliability and Agreement Studies) checklist (Kottner et al., 2011).

To measure the internal reliability of the questionnaire, McDonald's Omega coefficient was used, which was also applied to the five dimensions proposed as the initial model by the exploratory factor analysis (EFA).

To examine the construct validity of the dataset, an Exploratory Factor Analysis (EFA) was performed using the FACTOR program (version 12.06.08). The polychoric correlation matrix was employed, as the variables are of an ordinal nature. The number of factors to retain was determined by the Optimal Implementation of Parallel Analysis, which indicated a structure of five factors/dimensions. The extraction method utilized was Diagonally Weighted Least Squares (DWLS), which is suitable for ordinal and non-normal data, based on the polychoric matrix. A Promax oblique rotation was applied. To obtain more stable and reliable estimates, the Bootstrap procedure was used, generating 500 samples for resampling. 95 % confidence intervals were reported using the Bias-corrected and accelerated (BCa) method.

After conducting exploratory factor analysis (EFA), which allowed us to identify the underlying structure of the data, a confirmatory factor analysis (CFA) was performed to validate whether the factor structure proposed in the EFA adequately fits the collected data. In this analysis, we tested an initial hypothetical model that includes the relationships between the observed variables and the underlying factors. The lavaan package of the JASP program (version 0.95.4) was used. For parameter estimation, the Diagonally Weighted Least Squares (DWLS) estimator was selected because it is a method that utilizes the polychoric correlation matrix for the analysis and provides robust standard errors and fit indices in the presence of non-normality. Standardized weights were also included.

To evaluate model fit, several fit indices were considered, such as the Comparative Fit Index (CFI), the Tucker-Lewis Index (TLI), and the Root Mean Square Error of Approximation (RMSEA), among others.

### 3.3 Ethical considerations

Via electronic communication, Dr. Cox provided explicit consent to use and culturally adapt the IWPS-R into the Spanish (Castilian) language for this validation study, requesting only due acknowledgment of the original instrument.

Furthermore, in compliance with the methodological rigor for instrument adaptations that involve modifications (such as item exclusion), the research team is committed to sharing the final Spanish version (IWPS-R-ES) and the detailed documentation of all exclusion/modification decisions with Dr. Cox prior to its final dissemination.

The project was approved by the Ethics Committee of the Albacete Area Management and complied with all relevant ethical requirements, specifically those of anonymity and confidentiality.

The survey was conducted online, and the study population was accessed through corporate email using QR codes. Respect for the participants' autonomy and their right to confidentiality and anonymity was guaranteed, and all participants signed informed consent before participating in the study. The study was approved by the Drug Research Ethics Committee of Albacete and Valladolid.

The study was conducted in accordance with the principles of Law 14/2007 of July 3 on biomedical research, the standards of good practice, and the Declaration of Helsinki. The processing, communication, and transfer of all data was carried out in accordance with the provisions of Organic Law 3/2018, of December 5, on the protection of personal data and guarantees of digital rights. This law is the adaptation of the Spanish legal system to Regulation (EU) 2016/679 of the European Parliament and of the Council, of April 27, 2016, (GDPR).

All databases are blinded (no names, participant registration numbers, or any other identifying information that would link participants to their responses will be included) and will be safeguarded by the research team in accordance with current legislation. The research team will be the only people with access to them.

## 4 Results

The sample consisted of 305 nurses from various areas (hospitalization, emergency, critical care units, surgical block, outpatient clinics, day hospitals, etc.) working at the University Hospital Complex of Albacete (156) and the University Clinical Hospital of Valladolid (149). All respondents completed all items of the questionnaire, so no participants had to be excluded.

The demographic characteristics of the participants are presented in Table 1. The mean age was 44.14 years (SD 8.81), with 86.6 % (264) being women and 68.2 % (180) having at least one child in their care. 62.6 % (191) worked rotating shifts, while the median total work experience as nurses was 19 years (IQR 13).

**Table 1:** Comparison of demographic characteristics between participating hospitals.

		University Hospital Complex of Albacete N (156)	University Clinical Hospital of Valladolid N (149)	Statistical Test	*p*-value
Age (years) (Mean, SD)		44.23 (9.36)	44.04 (8.22)	*t* = 0.189^a^	0.851
Seniority in current job position (months) (Median, [IQR])		48 [84]	72 [96]	*U* = 8110.5^b^	<0.001
Total Labor Seniority (months) (Median, [IQR])		240 [156]	216 [144]	*U* = 10,834.0^b^	0.400
Sex (female) (n, %)		133(85.26)	131(87.92)	χ^2^=0.455^c^	0.500
Civil status (n, %)	Married	85 (54.48)	85 (57.04)	*F* = 5.272^d^	0.260
	Single	49 (31.41)	45 (30.20)		
	Divorced	16 (10.25)	9 (6.04)		
	Widowed	1 (0.64)	3 (2.01)		
	N/C	5 (3.20)	7 (4.69)		
Dependents (median IQR)		2 (2.25)	1 (2)	*U* = 10,563.0^b^	0.211
Job Contract Type (n, %)	Permanent contract	106 (67.94)	105 (70.46)	χ^2^=29.540^c^	<0.001
	Intern	18 (29.50)	43 (28.85)		
	Temporary contract	32 (20.51)	1 (0.67)		
Work Shift Regime (n, %)	Rotating shifts (AM-PM-N)	38(24.35)	76 (51.00)	χ^2^=115.820^c^	<0.001
	Fixed shifts (Monday to Friday)	36 (23.07)	52 (34.89)		
	Rotating shifts (Day-Night) 12 hr	76 (48.71)	1 (0.67)		
	Day shifts (AM/PM)	6 (3.84)	20 (13.42)		
Work department (n, %)	Hospitalisation	44 (28.20)	38 (25.50)	N/C	
	Critical care	30 (19.23)	18 (12.08)		
	Surgical block	14 (8.97)	23 (15.43)		
	Emergency	15 (9.61)	13 (8.72)		
	Outpatients	9 (5.76)	17 (11.04)		
	Day hospital	2 (1.28)	5 (3.35)		
	Hemodialysis	4 (2.56)	2 (1.34)		
	Cardiology/Vascular/Neurology Intervention	3 (1.92)	3 (2.01)		
	Laboratory	3 (1.92)	3 (2.01)		
	Radiotherapy	1 (0.64)	4 (2.68)		
	Birthing unit	3 (1.92)	1 (0.67)		
	Radiology	1 (0.64)	3 (2.01)		
	Pharmacy	2 (1.28)	1 (0.67)		
	Hematology	1 (0.64)	1 (0.67)		
	Preventive medicine	2 (1.2)	0		
	Quality unit	0	1 (0.67)		
	Other	22 (14.10)	16 (10.73)		

Note: ^a^ Student’s t-test

^b^ Mann-Whitney U test

^c^ Pearson’s Chi-square test (χ^2^).

^d^ Fisher's Exact Test; IQR: Interquartile Range; N/C: Not Categorized.

The two subsamples are homogeneous in fundamental variables such as Age (*p* = 0.851), Sex (*p* = 0.500), and total employment seniority (*p* = 0.400). This mitigates the risk of sampling bias associated with basic demographic characteristics. Regarding specific heterogeneity, significant differences were identified in three variables: seniority in the current position (*p* < 0.001), 48 vs. 72, which indicates a difference in staff stability in the specific position. In the type of contract (*p* < 0.001), there is a significant difference in the distribution of contract types. Hospital de Valladolid has a significantly higher proportion of Interim Statutory staff (28.86 % vs. 11.54 % in Albacete), while Albacete has more Temporary Statutory staff (20.51 % vs. 0.67 % in Valladolid). The shift pattern (*p* < 0.001) also differs significantly between the centers. The normality analysis for the 29 questionnaire items was performed using the Shapiro-Wilk test with a sample of *N* = 305 valid cases, showing negative skewness. The lack of normality in the items confirms the decision to use a robust method, such as the DWLS (Diagonally Weighted Least Squares) estimator, for the factor analysis. Detailed results of the normality analysis for all 29 items are provided in Supplementary Material.

### 4.1 Exploratory factorial analysis (EFA)

The Exploratory Factor Analysis (EFA) was carried out to examine the underlying factorial structure of the scale, using a sample of 305 participants and 29 variables. Before proceeding with the Exploratory Factor Analysis (EFA), the adequacy of the polychoric correlation matrix was evaluated. Bartlett’s Test of Sphericity was statistically significant (*p* < 0.001), and the Kaiser-Meyer-Olkin (KMO) measure of sampling adequacy yielded a value of 0.75, classified as acceptable, with a Bootstrap 95 % confidence interval of KMO = (0.093, 0.760). In the EFA, items 16, 17, and 20 did not meet the adequate factor loading criterion (<0.4) and were therefore excluded. In the Spanish version of the IWPS-R, 64.30 % of the total variance was explained by five extracted components. Appendix 1. For detailed information on the rotation matrix, please refer to the supplementary material.

The study identified a Spanish version composed of 26 items and five dimensions: Manager Support (F1) with eight items; Peer Support (F2) with six items; Organizational Resources (F3) with three items; Intention to stay (F4) with five items; and Workload (F5) with four items (Table 2). An omission criterion of (loadings lower than absolute 0.400 omitted) was used, meaning only factor loadings with an absolute value equal to or greater than 0.4 are shown.

**Table 2:** Rotated factor matrix. extraction method: DWLS. Rotation Method: Promax oblique.

	Factor/Dimension	F1 (Manager support)	F2 (Peer support)	F3 (Organizational resources)	F4 (Intention to stay)	F5 (Workload)
Q1	If the nurse manager is off duty, he unit is encouraged to contact her/him when there are staffing difficulties.	0.527				
Q2	If I complain about my workload to the nurse manager she/he will be empathetic.	0.833				
Q3	I stay in my current position because of the support of my nurse manager	0.594				
Q4	The nurse manager assists in working with patients and families who are unhappy with their care.	0.953				
Q5	The nurse manager is actively involved in securing enough staff each shift that is needed.	0.840				
Q6	The nurse manager actively works to fill open positions on the unit in a timely manner.	0.837				
Q7	My manager is competent in providing basic patient care in the unit.	0.951				
Q8	The charge nurse in my unit provides support for patient care when it is needed.	0.973				
Q9	I work with nurses whom I respect professionally.		0.660			
Q10	When I feel overwhelmed, I can count on other nurses to help me.		0.848			
Q11	The nurses work as a team.		0.888			
Q12	The nurses with whom I work are competent when caring for our typical patient population.		0.939			
Q13	I would feel comfortable having one of my family members cared for by staff on my unit.		0.658			
Q14	The nurses with whom I work are an important reason as to why I stay in my current job.		0.603			
Q15	Equipment (blood pressure machines, saturation monitors, scales, lifts, wheelchairs, thermometers) for patient care is available when I need it.			0.871		
Q18	Supplies (IV supplies, catheters, dressings, syringes, linens) for patient care are available when I need them.			0.972		
Q19	Pharmacy services provide adequate support in the medication process.			0.486		
Q21	My current workload will cause me to look for a new position.				0.464	
Q22	My current work environment makes me want to stay and work here.				−0.405	
Q23	I do not plan to stay in my current position for the next 12 months.				1.041	
Q24	I plan to stay in my current position for at least the next 12 months.				−0.881	
Q25	I intend to look for a new position in a different unit or in a different organization within the next 12 months.				1.005	
Q26	I am able to take at least a 30-minute meal break during my shift.					0.764
Q27	Individual assignments are fairly distributed within the unit given the available resources.					0.484
Q28	Most days I feel my workload is reasonable.					0.792
Q29	I have voiced concerns about my workload being too heavy to the nurse manager or charge nurse.					−0.460
ω de McDonald		0.908	0.827	0.773	0.880	0.706

### 4.2 Reliability

McDonald's Omega coefficient was calculated to assess the scale's reliability. The total reliability of the scale is good (*ω* = 0.88), with the subscales also being shown, where reliability is acceptable in dimensions F3 and F5, and good in the other three dimensions. Overall, the instrument proves to be reliable for the measurement of its constructs. See Table 3.

**Table 3:** Frequentist scale reliability statistics.

	Coefficient *ω*		95 % CI	
	Estimate	Std. Error	Lower	Upper
MODEL	0.882	0.009	0.865	0.899
F1	0.908	0.008	0.893	0.923
F2	0.827	0.016	0.798	0.857
F3	0.773	0.020	0.733	0.812
F4	0.880	0.011	0.861	0.900
F5	0.706	0.026	0.655	0.757

We included Cronbach's Alpha, which has a value of 0.814 (0.781–0.842), indicating good reliability consistent with ω. To detect weaknesses at the item level, we examined the item-rest correlation (r _i-t_) and alpha if the item is deleted (α _drop_). We found four items to be noted for their weakness.

Let us recall that in the exploratory analysis, we had already discarded three of the four items due to their low factor loading. Now a fourth problematic item appears, Q29, since its negative correlation (−0.166) and its impact on alpha (α _drop_ = 0.849) would justify its elimination. See appendix 2.

### 4.3 Confirmatory factorial analysis (CFA)

**Fig. 3:**
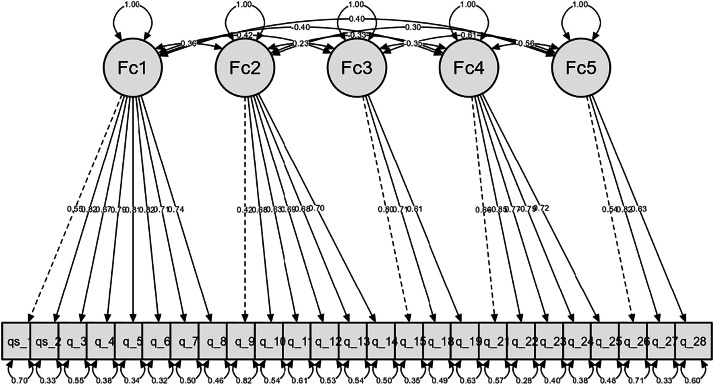
Five-dimension model of the IWPS-R-ES. Note: IWPS-R-ES = Revised individual workload perception scale – Spanish version.

We performed the Confirmatory Factor Analysis (CFA) for the five-dimension model of the IWPS-R-ES with the 26 proposed items, adjusted to a sample of 305 nurses. Standardized factor loadings were significant and exceeded 0.4 for all items, except for Item 29 (λ= −0.295). Since the standardized estimate does not reach the minimum threshold, we consider this item to be problematic, as it only shares a small part of the variance with the construct, it is supposed to measure and weakens convergent validity. Therefore, we decided to eliminate it to improve the convergent validity and reliability of the subscale (Factor/dimension 5). This adds further arguments to those presented in the previous point (item-rest correlation), so we consider its elimination justified. With Question 29 eliminated, we observe that all standardized loadings remain significant, and all items have standardized estimate values >0.4. See Fig. 3. Factor loading estimates for both the 25-item and 26-item models are provided in the supplementary material.

Regarding the relationship between each of the distinct dimensions, we observe the factor covariances, where all dimensions are significantly related (*p* < 0.001), both in a positive and negative manner. The matrix of correlations between factors is moderate (0.235 to 0.612), confirming that the factors are distinct constructs that operate together, which validates the choice of the first-order oblique model. See Appendix 3.

In Table 4, we can observe the percentage of explained variance absorbed by each of the items within each dimension.

**Table 4:** Individual item reliability and explained variance per factor (R^2^).

Factor	Explained Variance (*R*²)	Item	*R*²
F1	55.15 %	Q1	0.303
		Q2	0.665
		Q3	0.449
		Q4	0.622
		Q5	0.655
		Q6	0.676
		Q7	0.501
		Q8	0.541
F2	40.97 %	Q9	0.176
		Q10	0.461
		Q11	0.391
		Q12	0.473
		Q13	0.460
		Q14	0.497
F3	50.80 %	Q15	0.647
		Q18	0.508
		Q19	0.369
F4	47.44 %	Q21	0.434
		Q22	0.719
		Q23	0.597
		Q24	0.622
		Q25	0.524
F5	45.13 %	Q26	0.288
		Q27	0.666
		Q28	0.400

Note. *R*² = squared standardized factor loading. Items Q16, Q17, Q20, and Q29 were excluded from the final model due to insufficient factor loadings (< 0.40) or negative correlation with the scale.

The Convergent Validity of the instrument's dimensions was evaluated using the Average Variance Extracted (AVE), setting the acceptance threshold at AVE ≥ 0.50. Convergent Validity was adequate for Factors 1, 3, and 4 (> 0.530). However, insufficient convergent validity was identified in Factor 2 (AVE = 0.433) and Factor 5 (AVE = 0.431). Using these AVE values to test Discriminant Validity via the Fornell-Larcker Criterion, it was observed that all factors meet the criterion. Although the reliability (Omega Coefficient) for these factors was acceptable, AVE values below 0.50 suggest that, in these two dimensions, the error variance or non-construct-related variance explains a slightly greater proportion of item variance than the latent factor itself. Nevertheless, it was decided to retain these factors since Discriminant Validity (Fornell-Larcker Criterion) was confirmed for all constructs. See Appendix 4. The standardized factor loadings, standard errors, *p*-values, and explained variance (R^2^) for the retained items are detailed in Appendix 5. All items were statistically significant (*p* < 0.001). Overall, 92 % of the items met or exceeded the threshold for practical significance (λ≥0.50). After justifying the elimination of Item 29, the study identified a Spanish version composed of 25 items and five dimensions: Manager Support (F1) with eight items; Peer Support (F2) with six items; Organizational Resources (F3) with three items; Intention to Stay (F4) with five items; and Workload (F5) with three items. This is the IWPS-R-ES version. See Appendix 6. Regarding the overall fit indices, we can consider the proposed model adequate as the normalized chi-squared value is 1.595. See Table 5.

**Table 5:** Expected fit indices for a structural equation model and indices obtained for the AFC.

	*Chi-square test*				
Model		*Χ*²	df	p	Normalized Chi-squared (*χ*2/df)
Factor model		422.693	265	< .001	1595
		
	Metric				Value
Other fit measures	Root mean square error of approximation (RMSEA)				0.044
	Standardized root mean square residual (SRMR)				0.074
	Hoelter's critical N (α = 0.05)				219.615
	Hoelter's critical N (α = 0.01)				232.207
	Goodness of fit index (GFI)				0.958
	McDonald fit index (MFI)				0.772
	Expected cross validation index (ECVI)				1.785
Fit indices	Comparative Fit Index (CFI)				0.972
	Tucker-Lewis Index (TLI)				0.969
	Bentler-Bonett Non-normed Fit Index (NNFI)				0.969
	Bentler-Bonett Normed Fit Index (NFI)				0.929
	Parsimony Normed Fit Index (PNFI)				0.821
	Bollen's Relative Fit Index (RFI)				0.920
	Bollen's Incremental Fit Index (IFI)				0.972
	Relative Noncentrality Index (RNI)				0.972

Additional Goodness-of-Fit Measures Analyses were conducted. The Root Mean Square Error of Approximation (RMSEA=0.048) indicates that the approximation error is low, suggesting that the model fits the population well (recommended criterion < 0.08). The Standardized Root Mean Square Residual (SRMR = 0.074) value is acceptable as it shows that the average residual of the correlations is small. See Table 5. The Comparative Fit Indices were found to be above the 0.90 threshold: the Comparative Fit Index (CFI) was 0.972 and the Tucker-Lewis Index (TLI) was 0.969 (recommended criterion > 0.90). These values indicate that the proposed model is significantly superior to the null model, robustly explaining the observed covariance matrix.

Considering this analysis and the previous results (RMSEA=0.044, GFI=0.958, *χ*^2^/df=1.595), the overall conclusion regarding the Confirmatory Factor Analysis is positive. This means that the 5-factor model fits the observed data well.

The final proposed model is valid because it provides a satisfactory fit to the data. The proposed 5-dimensional model, along with its respective relationships, fits the data satisfactorily, indicating construct validity. We have factorial evidence that our instrument measures the perception of workloads in nurses, and that each of the indicators accurately measures its corresponding dimension of the instrument.

## 5 Discussion

This multicenter study was conducted across two tertiary care hospitals in different Spanish autonomous communities to evaluate the psychometric properties of the IWPS-R. Our validation retained a five-factor structure consistent with the original scale, although dimension naming was adapted to maximize conceptual validity within the Spanish nursing context.

We opted for 'Peer Support' instead of 'Team Support' to specifically reflect horizontal, peer-to-peer relationships between nurses. In the Spanish context, the term 'Team' often carries a broader, interdisciplinary connotation that might not accurately capture the specific support dynamic within the nursing group. Similarly, 'Organizational Resources' was chosen over 'Unit Support' because the items included—such as equipment and supplies—reflect resource allocation policies originating from managerial hospital structures rather than just the unit level. We acknowledge that these subtle nomenclature differences are a potential limitation for strict semantic comparability at a global level; however, prioritizing cultural validity ensures the scale reflects the reality of nurses in the Spanish National Health System (SNS).

The analysis of the factorial structure revealed a crucial item reduction. The 'Organizational Resources' (F3) dimension underwent the most significant reduction, retaining only three items (15, 18, 19). The exclusion of items 16 (social services), 17 (spiritual support), and 20 (psychological support) is justified by poor metric performance and limited relevance as direct workload sources within the SNS (Herrera Floro, 2019). While religious support is a valuable external aid for delegating spiritual and grief care, it does not directly mitigate the operational emergency workload managed by frontline nurses, explaining its low correlation with the main construct (Dueñas Pérez, 2024).

Item 20 (psychological/emotional support) showed a low correlation, suggesting it captures professional role frustration—perceived lack of time as a barrier to desired care—rather than a straightforward perception of workload. The elimination of these questions is consistent with other cultural adaptations, such as the Portuguese version, supporting the hypothesis that in the nursing culture of Southern Europe, these non-clinical factors have a lesser influence on workload perception (Cabrita et al., 2022). The reduction to three items in F3 resulted in a gain of factor, purity and precision, focusing directly on essential logistical and operational resources. Reliability for this factor remains in an acceptable range (*ω* > 0.70) confirming its validity.

In the 'Workload' (F5) dimension, Item 29 was excluded due to poor statistical performance and a negative item-total correlation. This item measures reporting behavior rather than perceived demand, making its variance inconsistent with the central construct. In the SNS, a strong hierarchy or a "culture of silence" regarding the belief that reporting is ineffective may convert this response into a factor of work culture or assertiveness, not workload perception (Hashemian et al., 2025). The retention of the remaining items (26, 27, 28) is adequate, as they measure the core operational workload: fairness in assignments, reasonableness of burden, and capacity for rest.

In line with international validations (Cabrita et al., 2022; Lin et al., 2011; Suliman et al., 2017), 'Manager Support' and 'Peer Support' demonstrated a greater influence than other dimensions. This global trend highlights the significant value nursing personnel place on supportive interactions with both supervisors and peers (Lin et al., 2011). Our findings coincide with validations in other countries regarding the five-factor structure, with only minor differences in specific items within the 'Organizational Resources,' 'Intention to Stay,' and 'Workload' dimensions.

## 6 Conclusions

The Spanish validation of the IWPS-R resulted in a robust 25-item instrument, the IWPS-R-ES. Factor analysis confirmed an adequate structure across five dimensions: Manager Support, Peer Support, Organizational Resources, Intent to Stay, and Workload. The scale demonstrated high internal reliability, establishing it as a valid and reliable tool for healthcare managers to assess nursing workload perceptions and inform evidence-based organizational decision-making.

## 7 Limitations

Despite the use of random probabilistic sampling, several limitations must be acknowledged. First, the sample was drawn from two tertiary hospitals in specific Spanish regions, which may limit the generalizability of findings to the entire Spanish National Health System (SNS). Second, the cross-sectional design precluded the evaluation of test-retest reliability, preventing confirmation of the temporal stability of latent factors. Finally, informing participants of the study objectives, in compliance with ethical standards, may have introduced social desirability bias.

## 8 Implications for practice

The IWPS-R-ES provides a rigorous and easily applicable tool for clinical management and research in Spanish-speaking environments. By quantifying individual perceptions of demands and resources, this instrument allows managers to identify critical stressors and implement targeted interventions. Ultimately, enhancing nurses' workload perceptions is a key lever for improving job satisfaction, staff retention (Intent to Stay), and patient safety.

## Data availability

The data that support the findings of this study are available on request from the corresponding author. The data are not publicly available due to privacy or ethical restrictions. To access data, readers may contact the author [jdfernandez@sescam.jccm.es]. An agreement of confidentiality is required to access certain sensitive data.

## CRediT authorship contribution statement

**Juan David Fernández-Sánchez:** Writing – review & editing, Writing – original draft, Validation, Supervision, Resources, Conceptualization. **Fernando Andrés-Pretel:** Methodology, Formal analysis, Data curation. **Inmaculada Pérez-Rodríguez:** Software, Project administration. **Milagros Molina-Alarcón:** Validation, Supervision. **Natan Redondo-Perez:** Writing – review & editing, Project administration. **Mercedes Fernández-Castro:** Writing – review & editing, Validation. **Cristina M. Lozano-Hernández:** Writing – review & editing, Validation, Supervision, Conceptualization.

## Declaration of competing interest

The authors declare that they have no known competing financial interests or personal relationships that could have appeared to influence the work reported in this paper.

## Figures and Tables

**Appendix 1:** Explained variance based on eigenvalues.

Variable	Eigenvalue	Proportion of Variance	Cumulative Proportion of Variance
Q1	906.878	0.31272	0.31272
Q2	336.156	0.11592	0.42863
Q3	275.046	0.09484	0.52348
Q4	202.877	0.06996	0.59343
Q5	143.841	0.04960	0.64303
Q6	124.097	0.04279	
Q7	0.99965	0.03447	
Q8	0.92646	0.03195	
Q9	0.81937	0.02825	
Q10	0.70917	0.02445	
Q11	0.62995	0.02172	
Q12	0.56689	0.01955	
Q13	0.52292	0.01803	
Q14	0.48943	0.01688	
Q15	0.44809	0.01545	
Q16	0.40447	0.01395	
Q17	0.35465	0.01223	
Q18	0.34573	0.01192	
Q19	0.33071	0.01140	
Q20	0.28262	0.00975	
Q21	0.25183	0.00868	
Q22	0.22211	0.00766	
Q23	0.19898	0.00686	
Q24	0.15825	0.00546	
Q25	0.13149	0.00453	
Q26	0.11398	0.00393	
Q27	0.09962	0.00344	
Q28	0.09461	0.00326	
Q29	0.01009	0.00035	

**Appendix 2:** Frequentist individual item reliability statistics.

Ítem	Corrected Item-Total Correlation	Cronbach's α if item deleted (α_drop_​)
Q16	0.264	0.817
Q17	0.201	0.820
Q20	0.312	0.811
Q29	−0.166	0.849

**Appendix 3:** Latent factor correlations and covariances*.*

							95 % Confidence Interval	
			Std. estimate	Std. Error	z-value	p	Lower	Upper
Factor 1	↔	Factor 2	0.358	0.066	5.423	< .001	0.228	0.487
Factor 1	↔	Factor 3	0.418	0.067	6.225	< .001	0.287	0.550
Factor 1	↔	Factor 4	−0.401	0.059	−6.833	< .001	−0.515	−0.286
Factor 1	↔	Factor 5	0.399	0.070	5.678	< .001	0.262	0.537
Factor 2	↔	Factor 3	0.235	0.065	3.593	< .001	0.107	0.363
Factor 2	↔	Factor 4	−0.331	0.065	−5.077	< .001	−0.459	−0.203
Factor 2	↔	Factor 5	0.304	0.068	4.483	< .001	0.171	0.437
Factor 3	↔	Factor 4	−0.351	0.063	−5.553	< .001	−0.475	−0.227
Factor 3	↔	Factor 5	0.612	0.064	9.542	< .001	0.486	0.737
Factor 4	↔	Factor 5	−0.560	0.060	−9.316	< .001	−0.678	−0.442

**Appendix 4:** Discriminant validity using the fornell-larcker criterion.

	Factor 1	Factor 2	Factor 3	Factor 4	Factor 5
Factor 1	746	358	418	401	399
Factor 2	358	658	235	331	304
Factor 3	418	235	728	351	612
Factor 4	401	331	351	755	560
Factor 5	399	304	612	560	656

**Appendix 5:** Factor loadings and practical significance.

Factor	Item (Indicator)	λ (Std. estimate)	Std. Error	Valor p	R^2^ (Explained Variance)	Practical Significance (λ≥0.50)
F1	question_1	0.551	0.062	<0.001	0.304	✓
	question_2	0.816	0.031	<0.001	0.666	✓
	question_3	0.670	0.044	<0.001	0.449	✓
	question_4	0.789	0.033	<0.001	0.622	✓
	question_5	0.810	0.032	<0.001	0.656	✓
	question_6	0.822	0.031	<0.001	0.676	✓
	question_7	0.708	0.036	<0.001	0.501	✓
	question_8	0.736	0.034	<0.001	0.542	✓
F2	question_9	0.420	0.076	<0.001	0.176	× No
	question_10	0.679	0.060	<0.001	0.461	✓
	question_11	0.625	0.057	<0.001	0.391	✓
	question_12	0.688	0.056	<0.001	0.473	✓
	question_13	0.678	0.061	<0.001	0.459	✓
	question_14	0.705	0.054	<0.001	0.497	✓ (Almost 0.50)
F3	question_15	0.804	0.054	<0.001	0.646	✓
	question_18	0.713	0.065	<0.001	0.508	✓
	question_19	0.608	0.069	<0.001	0.370	✓
F4	question_21	0.659	0.053	<0.001	0.434	✓
	question_22	(-)0.848	0.055	<0.001	0.719	✓
	question_23	0.772	0.041	<0.001	0.596	✓
	question_24	(-)0.789	0.042	<0.001	0.622	✓
	question_25	0.724	0.051	<0.001	0.524	✓
F5	question_26	0.537	0.061	<0.001	0.288	✓
	question_27	0.816	0.053	<0.001	0.666	✓
	question_28	0.633	0.054	<0.001	0.401	✓

**Appendix 6:** Original and back translation from Spanish versions of the individual workload perception scale-revised (IWPS-R).

Individual Workload Perception Scale (IWPS) – Revised		
Q.	Original	Spanish
	Think about your typical daily workload over the past six months and answer the following questions Scale:	Piense en su carga laboral diaria de los últimos seis meses y responda a las siguientes preguntas según esta escala:
	1= Strongly Disagree 2= Disagree 3= Unsure 4= Agree 5= Strongly Agree	1= Muy en desacuerdo 2= En desacuerdo 3= No estoy segura/o 4= De acuerdo 5= Muy en desacuerdo
Q1	If the nurse manager is off duty, he unit is encouraged to contact her/him when there are staffing difficulties.	Si la jefa/e de unidad de enfermería/supervisor/a no está de servicio, se posibilita que el personal pueda dirigirse al responsable a cargo en caso de incidencias en la plantilla o en el servicio.
Q2	If I complain about my workload to the nurse manager she/he will be empathetic.	Si me quejo de mi carga laboral a la jefa/e de unidad de enfermería/supervisor/a, muestra empatía.
Q3	I stay in my current position because of the support of my nurse manager	Sigo en mi puesto actual por el apoyo de mi jefa/e de unidad de enfermería/supervisor/a.
Q4	The nurse manager assists in working with patients and families who are unhappy with their care.	La jefa/e de unidad de enfermería/supervisor/a apoya o colabora en el trabajo con los pacientes y familias que no están satisfechos con los cuidados recibidos.
Q5	The nurse manager is actively involved in securing enough staff each shift that is needed.	La jefa/e de unidad de enfermería/supervisor/a se compromete activamente a garantizar la plantilla necesaria para cada turno.
Q6	The nurse manager actively works to fill open positions on the unit in a timely manner.	La jefa/e de unidad de enfermería/supervisor/a trabaja activamente para cubrir las ausencias del personal de la unidad en el plazo oportuno.
Q7	My manager is competent in providing basic patient care in the unit.	La jefa/e de unidad de enfermería/supervisor/a es competente para proporcionar cuidados de enfermería a los pacientes de la unidad.
Q8	The charge nurse in my unit provides support for patient care when it is needed.	La jefa/e de unidad de enfermería/supervisor/a proporciona apoyo en los cuidados a los pacientes cuando se necesita.
Q9	I work with nurses whom I respect professionally.	Respeto profesionalmente a las enfermeras o enfermeros con quienes trabajo.
Q10	When I feel overwhelmed, I can count on other nurses to help me.	Cuando siento que el trabajo me desborda, puedo contar con otras enfermeras/os para que me ayuden
Q11	The nurses work as a team.	Las enfermeras/os de mi unidad trabajan en equipo
Q12	The nurses with whom I work are competent when caring for our typical patient population.	Las enfermeras/os con quienes trabajo son competentes en el cuidado de los pacientes de mi unidad.
Q13	I would feel comfortable having one of my family members cared for by staff on my unit.	Me sentiría tranquila/o si alguno de mis familiares se encontrara al cuidado de cualquier miembro de mi unidad.
Q14	The nurses with whom I work are an important reason as to why I stay in my current job.	Las enfermeras/os con quienes trabajo son una razón importante por la que permanezco en mi puesto actual
Q15	Equipment (blood pressure machines, saturation monitors, scales, lifts, wheelchairs, thermometers) for patient care is available when I need it.	Dispongo del equipamiento necesario para el cuidado del paciente (tensiómetros, pulsioxímetros, básculas, grúas eléctricas, sillas de ruedas, termómetros) cuando lo necesito.
Q16	When the patients in my unit need them, the social service specialists are present in the hospital.	Cuando los pacientes de mi unidad necesitan la asistencia de servicios sociales, estos profesionales están disponibles en el hospital.
Q17	When a patient experiences a major crisis (code blue, new lifethreatening diagnosis) or dies a chaplain is available to support the patient and/or their family.	Cuando un/a paciente se encuentra en una situación crítica o muere, hay servicio espiritual disponible para asistir al paciente o a familiares.
Q18	Supplies (IV supplies, catheters, dressings, syringes, linens) for patient care are available when I need them.	Dispongo del material necesario para el cuidado del paciente (material para uso intravenoso, catéteres, vendas, jeringas, sábanas) cuando lo necesito.
Q19	Pharmacy services provide adequate support in the medication process.	El servicio de farmacia proporciona una asistencia adecuada en el proceso de la medicación.
Q20	I am able to provide adequate psychological /emotional support to the patients assigned to me.	Soy capaz de proporcionar un adecuado apoyo emocional o psicológico a los pacientes a mi cargo.
Q21	My current workload will cause me to look for a new position.	Mi carga laboral actual me obligará a buscar un nuevo puesto.
Q22	My current work environment makes me want to stay and work here.	Mi ambiente de trabajo me hace que quiera quedarme a trabajar aquí.
Q23	I do not plan to stay in my current position for the next 12 months.	No tengo planeado quedarme en mi puesto actual otros doce meses
Q24	I plan to stay in my current position for at least the next 12 months.	Tengo planeado quedarme en mi puesto actual al menos otros doce meses.
Q25	I intend to look for a new position in a different unit or in a different organization within the next 12 months.	Tengo la intención de buscar un nuevo puesto en otra unidad u organización diferente en los próximos doce meses.
Q26	I am able to take at least a 30-minute meal break during my shift.	Me puedo tomar al menos una pausa de treinta minutos durante mi turno.
Q27	Individual assignments are fairly distributed within the unit given the available resources.	Las tareas individuales se distribuyen de manera justa en la unidad dados los recursos disponibles.
Q28	Most days I feel my workload is reasonable.	La mayoría de los días siento que mi carga laboral es razonable.
Q29	I have voiced concerns about my workload being too heavy to the nurse manager or charge nurse.	He informado de mis preocupaciones por la gran carga de laboral que padezco a mi jefa/e de unidad de enfermería/supervisor/a.

## Appendix B Supplementary materials

Image, application 1
