# Supplementary material for: Cross-cultural adaptation and psychometric evaluation of the individual workload perception scale-revised (IWPS-R) in Spanish nurses
Source: Int J Nurs Stud Adv. 2026 Mar 3;10:100517. doi: 10.1016/j.ijnsa.2026.100517 (PMC12996835; doi:10.1016/j.ijnsa.2026.100517)
Supplement: Supplementary file 1 [file mmc1.pdf]

Supplementary material. Table 1. Descriptive Statistics.

	Valid	Missing	Median	IQR	Shapiro-Wilk	P-value
Q1	305	0	4.000	1.000	0.755	< .001
Q2	305	0	4.000	1.000	0.864	< .001
Q3	305	0	3.000	2.000	0.909	< .001
Q4	305	0	4.000	2.000	0.871	< .001
Q5	305	0	4.000	1.000	0.811	< .001
Q6	305	0	4.000	1.000	0.803	< .001
Q7	305	0	4.000	2.000	0.824	< .001
Q8	305	0	4.000	2.000	0.873	< .001
Q9	305	0	5.000	1.000	0.566	< .001
Q10	305	0	4.000	1.000	0.751	< .001
Q11	305	0	4.000	1.000	0.779	< .001
Q12	305	0	5.000	1.000	0.732	< .001
Q13	305	0	4.000	1.000	0.826	< .001
Q14	305	0	4.000	2.000	0.829	< .001
Q15	305	0	4.000	1.000	0.866	< .001
Q16	305	0	4.000	1.000	0.881	< .001
Q17	305	0	4.000	2.000	0.844	< .001
Q18	305	0	4.000	1.000	0.826	< .001
Q19	305	0	4.000	1.000	0.853	< .001
Q20	305	0	4.000	1.000	0.833	< .001
Q21	305	0	3.000	2.000	0.906	< .001
Q22	305	0	4.000	1.000	0.874	< .001
Q23	305	0	2.000	2.000	0.870	< .001
Q24	305	0	4.000	2.000	0.848	< .001
Q25	305	0	2.000	2.000	0.867	< .001
Q26	305	0	3.000	2.000	0.898	< .001
Q27	305	0	4.000	1.000	0.856	< .001
Q28	305	0	3.000	2.000	0.890	< .001
Q29	305	0	4.000	1.000	0.869	< .001

Supplementary material. Table 3. Rotated loading matrix.

Variable	F 1	F 2	F 3	F 4	F 5
question_1	0.527	0.110	0.019	0.075	0.077
question_2	0.833	-0.025	0.013	-0.008	0.025
question_3	0.594	-0.055	0.054	-0.114	0.008
question_4	0.953	-0.060	0.030	-0.011	-0.079
question_5	0.840	0.011	-0.049	0.092	0.234
question_6	0.837	-0.021	-0.002	0.037	0.160
question_7	0.951	-0.034	-0.076	-0.041	-0.135
question_8	0.973	-0.014	0.024	-0.036	-0.204
question_9	0.032	0.660	-0.093	0.035	-0.027
question_10	0.008	0.848	-0.117	0.045	0.159
question_11	-0.113	0.888	0.025	0.011	-0.059
question_12	0.002	0.939	-0.028	0.052	-0.047
question_13	-0.035	0.658	0.096	-0.036	0.049
question_14	0.053	0.603	0.128	-0.173	-0.287
question_15	0.052	-0.097	0.871	0.019	-0.008
question_16	0.031	0.133	0.280	0.001	-0.043
question_17	0.138	0.064	0.225	-0.076	-0.108
question_18	-0.032	-0.032	0.972	0.051	-0.051
question_19	-0.003	0.033	0.486	0.056	0.230
question_20	0.115	0.111	0.276	-0.074	0.024
question_21	0.008	0.052	-0.035	0.464	-0.443
question_22	0.118	0.328	0.057	-0.405	0.101
question_23	0.013	0.026	0.033	1.041	0.015
question_24	0.026	0.022	-0.002	-0.881	0.013
question_25	-0.010	0.026	0.062	1.005	0.010
question_26	0.062	0.029	-0.078	0.062	0.764
question_27	0.175	0.304	0.083	0.060	0.484
question_28	-0.077	-0.109	0.100	-0.153	0.792
question_29	0.127	0.140	0.033	0.177	-0.460

Supplementary material. Table 3. Factor loading parameter estimates. 26 items,

						95% Confidence Interval	
Factor	Indicator	Std. estimate	Std. Error	z-value	p	Lower	Upper
Factor 1	Q1	0.549	0.063	8.761	< .001	0.426	0.671
	Q2	0.817	0.031	26.374	< .001	0.756	0.878
	Q3	0.670	0.044	15.277	< .001	0.584	0.756
	Q4	0.788	0.033	23.925	< .001	0.723	0.852
	Q5	0.810	0.032	25.278	< .001	0.747	0.873
	Q6	0.824	0.031	26.752	< .001	0.764	0.884
	Q7	0.708	0.036	19.439	< .001	0.636	0.779
	Q8	0.735	0.035	21.270	< .001	0.667	0.803
Factor 2	Q9	0.419	0.076	5.545	< .001	0.271	0.567
	Q10	0.679	0.061	11.197	< .001	0.560	0.797
	Q11	0.624	0.057	10.856	< .001	0.511	0.736
	Q12	0.688	0.057	12.147	< .001	0.577	0.799
	Q13	0.678	0.061	11.051	< .001	0.558	0.799
	Q14	0.707	0.057	12.453	< .001	0.596	0.818
Factor 3	Q15	0.803	0.054	14.782	< .001	0.696	0.909
	Q18	0.712	0.065	10.900	< .001	0.584	0.840
	Q19	0.610	0.070	8.766	< .001	0.473	0.746
Factor 4	Q21	0.668	0.052	12.915	< .001	0.567	0.770
	Q22	-0.838	0.055	-15.313	< .001	-0.945	-0.730
	Q23	0.774	0.040	19.500	< .001	0.696	0.852
	Q24	-0.790	0.042	-18.959	< .001	-0.872	-0.708
	Q25	0.724	0.051	14.055	< .001	0.623	0.825
Factor 5	Q26	0.561	0.059	9.446	< .001	0.445	0.678
	Q27	0.806	0.054	14.810	< .001	0.700	0.913
	Q28	0.687	0.050	13.676	< .001	0.589	0.785
	Q29	-0.295	0.071	-4.141	< .001	-0.434	-0.155

Supplementary material. Factor loading parameter estimates. 25 items, Q29 removed.

						95% Confidence	
Factor	Indicator	Std. estimate	Std. Error	z-value	p	Lower	Upper
Factor 1	Q1	0.551	0.062	8.873	< .001	0.429	0.672
	Q2	0.816	0.031	26.341	< .001	0.755	0.876
	Q3	0.670	0.044	15.260	< .001	0.584	0.756
	Q4	0.789	0.033	23.874	< .001	0.724	0.853
	Q5	0.810	0.032	25.486	< .001	0.747	0.872
	Q6	0.822	0.031	26.648	< .001	0.762	0.883
	Q7	0.708	0.036	19.487	< .001	0.636	0.779
	Q8	0.736	0.034	21.452	< .001	0.669	0.803
Factor 2	Q9	0.420	0.076	5.554	< .001	0.272	0.568
	Q10	0.679	0.060	11.229	< .001	0.561	0.798
	Q11	0.625	0.057	10.911	< .001	0.513	0.738

	Q12	0.688	0.056	12.225	< .001	0.578	0.798
	Q13	0.678	0.061	11.128	< .001	0.559	0.798
	Q14	0.705	0.056	12.479	< .001	0.594	0.816
Factor 3	Q15	0.804	0.054	14.817	< .001	0.698	0.911
	Q18	0.713	0.065	10.996	< .001	0.586	0.840
	Q19	0.608	0.069	8.767	< .001	0.472	0.744
Factor 4	Q21	0.659	0.053	12.447	< .001	0.555	0.763
	Q22	-0.848	0.055	-15.480	< .001	-0.955	-0.741
	Q23	0.772	0.041	18.901	< .001	0.692	0.852
	Q24	-0.789	0.042	-18.625	< .001	-0.872	-0.706
	Q25	0.724	0.051	14.155	< .001	0.623	0.824
Factor 5	Q26	0.537	0.061	8.866	< .001	0.418	0.655
	Q27	0.816	0.053	15.366	< .001	0.712	0.920
	Q28	0.633	0.054	11.807	< .001	0.528	0.738
